# The Signatures of Natural Selection and Molecular Evolution in Fusarium graminearum Virus 1

**DOI:** 10.3389/fmicb.2020.600775

**Published:** 2020-11-12

**Authors:** Jeong-In Heo, Jisuk Yu, Hoseong Choi, Kook-Hyung Kim

**Affiliations:** ^1^Department of Agricultural Biotechnology, College of Agriculture and Life Sciences, Seoul National University, Seoul, South Korea; ^2^Plant Genomics and Breeding Institute, Seoul National University, Seoul, South Korea; ^3^Research Institute of Agriculture and Life Sciences, Seoul National University, Seoul, South Korea

**Keywords:** FgV1, positive selection, deleterious mutation, viral fitness, serial passage

## Abstract

Fusarium graminearum virus 1 (FgV1) is a positive-sense ssRNA virus that confers hypovirulence in its fungal host, *Fusarium graminearum*. Like most mycoviruses, FgV1 exists in fungal cells, lacks an extracellular life cycle, and is therefore transmitted during sporulation or hyphal anastomosis. To understand FgV1 evolution and/or adaptation, we conducted mutation accumulation (MA) experiments by serial passage of FgV1 alone or with FgV2, 3, or 4 in *F. graminearum*. We expected that the effects of positive selection would be highly limited because of repeated bottleneck events. To determine whether selection on the virus was positive, negative, or neutral, we assessed both the phenotypic traits of the host fungus and the RNA sequences of FgV1. We inferred that there was positive selection on beneficial mutations in FgV1 based on the ratio of non-synonymous to synonymous substitutions (*d_*N*_/d_*S*_*), on the ratio of radical to conservation amino acid replacements (*p_*NR*_/p_*NC*_*), and by changes in the predicted protein structures. In support of this inference, we found evidence of positive selection only in the open reading frame 4 (ORF4) protein of DK21/FgV1 (MA line 1); mutations at amino acids 163A and 289H in the ORF4 of MA line 1 affected the entire structure of the protein predicted to be under positive selection. We also found, however, that deleterious mutations were a major driving force in viral evolution during serial passages. Linear relationships between changes in viral fitness and the number of mutations in each MA line demonstrated that some deleterious mutations resulted in fitness decline. Several mutations in MA line 1 were not shared with any of the other four MA lines (PH-1/FgV1, PH-1/FgV1 + 2, PH-1/FgV1 + 3, and PH-1/FgV1 + 4). This suggests that evolutionary pathways of the virus could differ with respect to hosts and also with respect to co-infecting viruses. The data also suggested that the differences among MA lines might also be explained by mutational robustness and other unidentified factors. Additional research is needed to clarify the effects of virus co-infection on the adaptation or evolution of FgV1 to its environments.

## Introduction

Because they have small genomes and high mutation rates, RNA viruses usually form populations with high genetic variation. Such viral populations, which are known as quasispecies, maintain the balance between the continuous generation of mutations and the natural selection that acts on the mutants in relation to their fitness (Domingo and Holland, 1997). Researchers have therefore tried to understand the various evolutionary processes that shape the structure and fitness of viral populations. In this regard, mutation accumulation (MA) experiments have been conducted with several host–virus systems to determine the effects of spontaneous mutations on viral fitness (Elena et al., 2006). When viruses undergo consecutive transmission within their hosts, the effects of mutations arise in different ways depending on the size of the population (Domingo and Holland, 1997). If large population sizes are maintained, deleterious mutations are purged by natural selection, and beneficial mutations steadily increase in frequency over time to increase fitness. However, if a population experiences increased genetic drift under repeated bottleneck events during transmission, i.e., plaque-to-plaque transfer, fitness gradually decreases with the accumulation of deleterious mutations; this process is termed Muller’s ratchet (Domingo and Holland, 1997; McCrone and Lauring, 2018).

Muller’s ratchet has been widely studied via MA experiments for many RNA viruses that infect bacteria, animals, and plants (Elena et al., 2006). A study of bacteriophage φ6, for which this phenomenon was first demonstrated, revealed that the fitness of the virus was reduced by an average of 22% after 40 bottleneck passages as the result of plaque-to-plaque transfers (Chao, 1990). Similarly, the animal pathogens vesicular stomatitis virus, foot and mouth disease virus, and human immunodeficiency virus type 1 showed 38%, 35%, and 82% fitness loss after 20, 30, and 15 bottleneck passages, respectively (Duarte et al., 1992; Escarmís et al., 1996; Yuste et al., 1999). Among plant RNA viruses, tobacco etch virus experienced a 5% decline in fitness per passage for up to 11 passages (De la Iglesia and Elena, 2007). Although a few lineages experienced an increase in fitness, fitness decline has been the dominant phenomenon in various MA experiments with RNA viruses. Such sensitivity of RNA viruses to deleterious mutations (caused by their highly unstable replication systems along with the compactness of their genomes and the overlapping functions of their non-redundant genes) suggests that the fitness of RNA viruses will be dominated by purifying selection of deleterious mutations.

To date, four RNA mycoviruses (Fusarium graminearum virus 1–4, hereafter referred to as FgV1–4) of the plant-pathogenic fungus *Fusarium graminearum* have been discovered in South Korea (Chu et al., 2002, 2004; Cho et al., 2013). FgV1–4 were assigned to four families (Kwon et al., 2007; Yu et al., 2009, 2011; Li et al., 2019). We previously adapted FgV1–4 to the laboratory host, *F. graminearum* strain PH-1, in order to investigate the effects of virus infection on the biological characteristics and transcriptional alterations of the fungus (Lee et al., 2014). FgV1 and 2 cause hypovirulence, while FgV3 and 4 cause asymptomatic infection in their fungal host (Cho et al., 2013; Lee et al., 2014). Host transcriptome analysis revealed that phenotypic differences in PH-1were not always correlated with differential gene expression caused by FgV1–4 infections (Lee et al., 2014). Moreover, recent studies demonstrated that the transcriptional response of host RNA interference (RNAi)-related genes to infection by FgV1, FgV2, or FgV3 differed depending on which virus infected the host, and that FgV1 can interfere with the induction of RNAi-related genes to counteract host antiviral defense responses (Yu et al., 2018, 2020). However, researchers have yet to determine how these viruses adapt to their host and continuously maintain their own characteristics.

FgV1–4 were originally isolated from different host strains, i.e., FgV1 was isolated from *F. graminearum* strain DK21 (*F. boothii*; lineage 3), FgV2 from strain 98-8-60 (*F. asiaticum*; lineage 6), and FgV3 and FgV4 from strain DK3 (*F. graminearum sensu stricto*; lineage 7) (Lee et al., 2014). In addition, two viruses that share high sequence similarity with FgV1 or FgV2 have been recently isolated in China (hereafter referred to as FgV1-ch and FgV-ch9, respectively) (Zhang et al., 2020). Considering that the original host strains of FgV1–4 belong to different lineages in the *Fusarium graminearum* species complex (FGSG), a complex that includes eight and several additional phylogenetic lineages that cause Fusarium head blight (FHB), the potential of adaptive evolution in FgVs can be inferred (Amarasinghe et al., 2019). In this regard, the following question should be answered: Which evolutionary processes dominate FgV1 populations and consequently shape the structure and fitness of the populations?

Here, we conducted MA experiments to detect and quantify natural selection on FgV1 at the molecular level. We also introduced FgV2, FgV3, or FgV4 into FgV1-infected hosts to observe the effects of passages on FgV1 and to understand the changes in viral fitness caused by interactions between co-infecting viruses.

## Materials and Methods

### Virus Infections and Fungal Cultures

All fungal host strains used in this study (Table 1; Lee et al., 2014) were stored in 25% (v/v) glycerol at −80°C and were reactivated on potato dextrose agar (PDA; Difco) plates. Fungal hosts infected with two viruses (PH-1/FgV1 + FgV2, 3, or 4) were obtained through hyphal anastomosis on PDA agar medium. Mycelial plugs taken from the interface region between two isolates infected respectively with FgV1 and FgV2, 3, or 4 (PH-1/FgV1 and PH-1/FgV2, 3, or 4) were subcultured on PDA medium. In case of FgV1 and 2, infection of each isolate grown from the mycelial plugs was confirmed by dsRNA profiles on 0.8% agarose gel after S1 nuclease and DNase I treatment, while in case of FgV3 and 4, infection was confirmed by a semi-quantitative RT-PCR. Fungal colonies were subcultured on complete medium (CM) agar plates and were incubated at 25°C. After infection by FgV1 was confirmed based on phenotype, each subculture was cultured in 50 ml of liquid CM at 25°C on an orbital shaker (150 rpm) for 5 days. Mycelia were harvested by filtration through Whatman 3MM filter paper, and were then washed with distilled water, pressed between paper towels to remove the excess water, and stored at −80°C (Lee et al., 2014). Stored mycelia were used for further experiments.

**TABLE 1:** MA lines subjected to serial passage.

MA line	Host strain	Virus infection	Generations selected for RNA-Seq
1	DK21	FgV1 (Single infection)	1^st^, 5^th^, 10^th^, and 15^th^
2	PH-1	FgV1 (Single infection)	1^st^, 5^th^, 10^th^, and 15^th^
3	PH-1	FgV1 and FgV2 (Multiple infection)	2^nd^, 6^th^, 11^th^, and 12^th^
4	PH-1	FgV1 and FgV3 (Multiple infection)	2^nd^, 6^th^, 11^th^, and 16^th^
5	PH-1	FgV1 and FgV4 (Multiple infection)	2^nd^, 6^th^, 11^th^, and 12^th^

### Total RNA Extraction, dsRNA Isolation, and Reverse Transcription-Polymerase Chain Reaction (RT-PCR)

Total RNA was extracted with RNAiso Plus (Takara Bio Inc.) according to the manufacturer’s instructions with slight modifications, and was treated with DNase I (Takara Bio Inc.) to eliminate genomic DNA. RNA was precipitated by ethanol with 3M NaOAc and was suspended in DEPC-treated water and then examined by agarose gel (0.8%) electrophoresis. Due to low viral titers in FgV3 or FgV4 infected fungal strains, we performed RT-PCR with virus specific primer to determine existence of virus. To detect FgV3 and 4 infections, cDNAs were synthesized from 5 μg of RNA with GoScript reverse transcriptase (Promega) followed by PCR reactions with virus-specific primer sets for FgV3 and 4 (Supplementary Table S1). PCR reactions used synthesized cDNAs to detect the viruses and were performed with the following conditions for FgV3: one step at 94°C for 3 min; followed by 30 cycles at 94°C for 20 s, 58°C for 30 s, and 72°C for 30 s; and a final step at 72°C for 10 min. In the case of FgV4, the PCR conditions included one step at 94°C for 3 min; followed by 35 cycles at 94°C for 25 s, 58°C for 40 s, and 72°C for 30 s; and a final one step at 72°C for 10 min. Amplified PCR products were analyzed by 1.2% agarose gel electrophoresis. To detect FgV1 and 2, generally have high viral titers in fungal strains, dsRNAs were isolated from total RNA through S1 nuclease and DNase I treatment and were analyzed by 0.8% agarose gel electrophoresis.

### Serial Passages in Fungal Hosts

Experimental evolution of FgV1 was promoted by consecutive vertical transmissions in two hosts, *F. graminearum* strains DK21 and PH-1. Vertical transmission of viruses was conducted by serial passaging in the FgV1-host (without or with other viruses, as indicated in Table 1), which included the following steps: (1) conidiation of the host in carboxymethyl cellulose (CMC) liquid medium; (2) isolation of single conidia infected by virus(es) in different MA lines (Table 1); and (3) confirmation of virus infection in each single isolate. In brief, mycelial blocks were placed in CMC and incubated at 25°C and 150 rpm for 5 days. In the next step, diluted conidial suspensions containing approximately 100 conidia per mL were spread on CM agar plates, and the plates were incubated at 25°C for at least 24 h until the conidia germinated. After single conidia were transferred to CM agar plates, infection of FgV1 was first confirmed by the phenotype of the fungal colony produced by each conidium. Each colony was placed in liquid CM to obtain mycelia for total RNA extraction. Co-infection by FgV1 and 2 was confirmed by dsRNA profiles on 0.8% agarose gel after S1 nuclease and DNase I treatment. Co-infection by FgV1 and 3 and by FgV1 and 4 was confirmed by a semi-quantitative RT-PCR. Among isolates for which single infections (MA lines 1 and 2) or multiple infections (lines 3, 4, and 5) were confirmed, three isolates were randomly chosen per line and placed on CMC to obtain conidia of the next generation.

### Extraction of Total RNA and RNA-Sequencing Analysis

For RNA-sequencing, fungal isolates were grown, harvested, and stored as described in the previous section. Three single conidial isolates, where single or co-infection was confirmed by the aforementioned methods, were randomly chosen from each generation of each line (three biological replicates), and used for RNA-sequencing. After each isolate was cultured three times and harvested separately, total RNA was extracted from each of the three replicate cultures (three technical replicates), mixed to constitute one total RNA sample of one isolate, and used for RNA-Sequencing. Total RNAs were extracted from ground mycelia using the easy-spin^TM^ Total RNA Extraction Kit (iNtRON Biotechnology) according to the manufacturer’s instructions. Extracted total RNAs were treated with DNase I (Takara Bio Inc.) to eliminate genomic DNA. Pair-end mRNA libraries were generated using the TruSeq^TM^ Stranded mRNA kit and were sequenced using Illumina NovaSeq6000 platforms at Macrogen (Seoul, South Korea).

### Analysis of SNPs of FgV1 Using Transcriptome Data

SNP analysis for FgV1 was conducted as previously described (Jo et al., 2018). In the current study, we mapped the raw sequence reads for each transcriptome to the complete sequence of FgV1 (NC_006937.2) using the BWA program. The SAM files obtained by BWA mapping were converted to BAM files using SAMtools (Li et al., 2009). With the mpileup function of SAMtools, we generated VCF files from the sorted BAM files. Finally, we applied BCFtools to identify the SNPs.

### Statistical Analyses

Sequences of each open reading frame (ORF) containing SNPs were aligned using MUSCLE software^1^ (Okonechnikov et al., 2012), and phylogenetic trees were reconstructed with the neighbor-joining method implemented with Unipro UGENE software^2^ (Okonechnikov et al., 2012). With the multiple sequence alignments obtained as described above, the site-specific models (Nielsen and Krogh, 1998) that allow the *d_*N*_/d_*S*_* (hereafter referred to as ω) ratio to vary among sites but not among lineages were implemented by CodeML in the PAML (Phylogenetic Analysis by Maximum Likelihood) package of programs (Yang, 1997). After observed data were fit under models M1 (nearly neutral model), M2 (positive selection model), M7 (beta), and M8 (beta and omega > 1), the goodness-of-fit of each model was determined by calculation of log-likelihoods. Based on log-likelihoods, M1 with two classes of codons (ω = 0 for conserved sites and ω = 1 for neutral sites) was compared to M2 with an additional class of codons with ω estimated from the data allowed to be greater than 1. Likewise, M7 with ω ratios under a beta distribution was compared to M8 with an additional class of codons with ω allowed to be greater than 1 (Swanson and Aquadro, 2002). Statistically significant evidence of positive selection was inferred by a likelihood ratio test (LRT) that compared twice the log-likelihood difference (2ΔlnL) between two models which is assumed to be under the χ^2^ distribution with df = 2 (Wang et al., 2014). The null hypothesis was that a simpler model with no consideration of positive selection, i.e., M1 or M2, best fit the observed data. If the null hypothesis was rejected by LRTs, we concluded that M2 or M8 fit the observed data significantly better than M1 or M7; this would indicate that there was positive selection among compared sequences.

To calculate *pNR/pNC* ratios, all possible amino acid replacements were classified according to two widely used criteria, i.e., charge and polarity. The ratio of the number of radical amino acid replacements per radical non-synonymous site, the proportion of radical non-synonymous substitutions (*pNR*) and the number of conservative amino acid replacement changes per conservative non-synonymous site, and the proportion of conservative non-synonymous substitutions (*pNC*) were calculated with the HON-NEW program^3^, which uses a modification of the previous method (Hughes et al., 1990) by taking the transition bias into account.

In a second method to assess positive selection, we examined the overall tendency in the type of amino acid replacements caused by non-synonymous mutations. Such a tendency is determined by the *pNR/pNC* ratio, i.e., the ratio of the number of radical amino acid replacements per radical non-synonymous site (the proportion of radical non-synonymous substitutions; *pNR*) to the number of conservative amino acid replacement changes per conservative non-synonymous site (the proportion of conservative non-synonymous substitutions; *pNC*). When *pNR* = *pNC*, we can infer that amino acid replacements take place at random with respect to the property of amino acids and that there is no selection under neutral evolution. When *pNR* < *pNC*, the replacements conserve the property under purifying selection. However, when *pNR* > *pNC*, the replacements promote radical changes in the property under positive selection (Swanson and Aquadro, 2002).

### Prediction of Protein Structures

The structures of proteins from sequences with different combinations of mutations found in each replicate were predicted using the I-TASSER Server^4^ and were compared to the wild type and to one another using the PyMOL program^5^.

## Results

### Changes in Biological Traits During Passages

**FIGURE 1:**
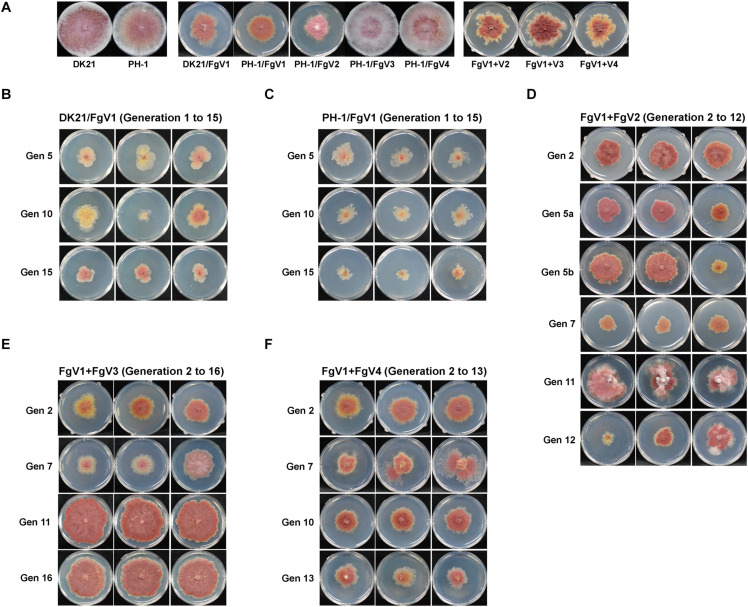
Colony morphologies throughout passaging. **(A)** Colony morphology of virus-free and virus-infected fungal strains used for single conidia isolation in this study. Cultures were photographed after 5 days on CM. DK21/FgV1 (**B**; MA line 1), PH-1/FgV1 (**C**; MA line 2), PH-1/FgV1 + 2 (**D**; MA line 3), PH-1/FgV1 + 3 (**E**; MA line 4), and PH-1/FgV1 + 4 (**F**; MA line 5). **(B,C)** Gen 5, 10, and 15 indicate the 5th, 10^th^, and 15^th^ generations, respectively. **(D)** Gen 5a shows morphologies of isolates infected with FgV1 + 2 in the 5^th^ generation of MA line 3, while 5b shows the morphologies of isolates infected singly with FgV1, i.e., with no FgV2 transmitted from the previous generation. For Gen 12 of those isolates originally infected with FgV1 + 2, the two cultures on the left were infected only with FgV1, while the culture on the right was infected with both FgV1 and FgV2. **(B–F)** All cultures in were photographed 4 days after transfer of single conidia, except cultures of Gen 7 **(E)** were photographed 2 days after transfer to show their fast growth.

As shown in Table 1, passaging was conducted with isolates infected with a single virus (MA lines 1 and 2) or with two viruses (MA lines 3, 4, and 5) for at least 12 generations to observe the effect of spontaneous mutations. Passaging of MA lines 3 (PH-1/FgV1 + 2) and 5 (PH-1/FgV1 + 4) was conducted for 12 generations (the co-infection by two viruses was not maintained after the 12th generation), and replicates from the 2^nd^, 6^th^, 11^th^, and 12^th^ generations were selected. Because of the hypovirulence conferred by FgV1 on both host strains, the phenotypes of the host fungi in the 1st generation of the MA lines 3, 4, and 5 were indistinguishable from each other (Figure 1A). Throughout serial passaging up to the 15^th^, 12^th^, or 16^th^ generation (for lines 3, 5, and 4, respectively), the phenotypes of both host strains singly infected with FgV1 (MA lines 1 and 2) were generally maintained with a slight decrease in mycelial growth and with slight variation between replicates in each generation (Figures 1B,C). PH-1 strain infected with FgV1 + FgV2, 3, or 4 (MA lines 3, 4, and 5) exhibited a few differences relative to the other MA lines. In the case of MA line 3 (PH-1/FgV1 + 2) (Figure 1D), variation among replicates of the same generation were greater than for other MA lines. Observation of dsRNA accumulation among biological replicates in the same generation of MA line 3 showed variation of dsRNA accumulation (Supplementary Figure S1). Also, colony morphologies of virus-infected isolates in MA line 3 exhibited unstable and irregular growth in the 11^th^ and 12^th^ generations (Figure 1D). While the phenotype was more regular at earlier and later generation for MA line 5 (PH-1/FgV1 + 4) than for the singly infected line (Figures 1C,F), some isolates in MA line 4 (PH-1/FgV1 + 3) began to exhibit faster mycelial growth in the 7^th^ generation (Figure 1E) than those infected with FgV1. The biggest differences in the phenotype of fast-growing isolates compared to that of virus-free isolates included scarce aerial mycelia and increased pigmentation. From the 11^th^ generation of MA line 4, all the conidia spread on agar media grew into fast-growing isolates, and their changed phenotype was maintained until the last generation (Figure 1E).

FgV1 had a vertical transmission rate of 100% except for MA line 3 (PH-1/FgV1 + 2), for which the FgV1 vertical transmission rate decreased to 71% in the 11^th^ generation and to 75% in the 12^th^ generation (Table 2). However, there was no clear trend in the changes in vertical transmission rates for any of three other co-infected lines, but fluctuations in the transmission rate were substantial for FgV3 in co-infected MA line 4 (Table 2). Because the accumulation of FgV4 in co-infected MA line 5 was low according to semi-quantitative RT-PCR, FgV4 was not considered to have been transmitted to the last (13^th^) generation of MA line 5 (PH-1/FgV1 + 4) in the first replicate, which included 12 conidia (Supplementary Figure S2).

**TABLE 2:** Vertical transmission efficiency of FgV1–4.

Host strain	DK21	PH-1	PH-1					
Virus infection	FgV1	FgV1	FgV1 + FgV2		FgV1 + FgV3		FgV1 + FgV4	
MA line	1	2	3		4		5	
Generation^a^	FgV1	FgV1	FgV1	FgV2	FgV1	FgV3	FgV1	FgV4
1 (2)	1.00	1.00	1.00	0.96	1.00	0.83	1.00	0.96
5 (6)	1.00	1.00	1.00	0.96	1.00	0.75	1.00	0.71
10 (11)	1.00	1.00	0.71	0.86	1.00	0.83	1.00	0.58
15 (12 or 16)	1.00	1.00	0.75	1.00	1.00	0.21	1.00	0.46

*^*a*^Each value indicates the vertical transmission rate of each virus from the previous to the next generation. Generation numbers 1, 5, 10, and 15 apply to MA line 1 and 2, while generation numbers in parenthesis (2, 6, 11, 12, or 16) apply to MA line 3, 4, and 5.*

### RNA-Sequencing

As noted earlier, we conducted passaging up to the 12^th^ or 15^th^ generation on each MA line, and three replicates from each of the indicated generations were subjected to RNA-Sequencing. The percentage of the total viral reads obtained from all generations of MA lines was higher for FgV1 than for the other viral strains (Supplementary Table S2). Observation of higher accumulation level of FgV1 viral dsRNA compared to that of other FgVs in fungal host could support high proportion of viral reads in MA lines (Supplementary Figure S3). All of the transcriptomic data have been deposited in NCBI data base (Accession: PRJNA656941, Supplementary Table S3).

### Overview of Single-Nucleotide Polymorphisms

**FIGURE 2:**
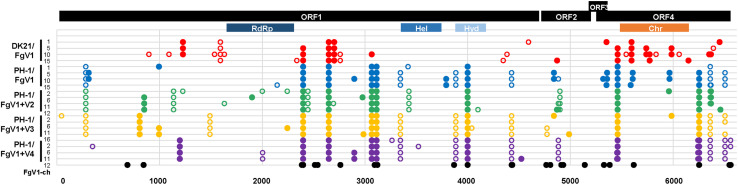
Overview of single-nucleotide polymorphisms. Closed and open circles, respectively, indicate non-synonymous and synonymous SNPs identified by statistical analyses. All SNPs found in each consensus sequence of FgV1 obtained from raw sequence data of the three biological replicates in each generation of each MA line were incorporated and shown together in each row. RdRp, RNA-dependent RNA polymerase-like family; Hel, DEAD-like helicases superfamily; Hyd, P-loop containing nucleoside triphosphate hydrolases; Chr, chromosome segregation protein (provisional), based on the NCBI database. [In case of FgV1-ch (Accession: MT024571.1), only non-synonymous substitutions identified by the sequence alignment of FgV-ch and the reference sequence of FgV1 in the NCBI database are shown].

We first focused on the patterns of SNPs in the whole genome of FgV1 that differed with the host strains. We could not find a specific insertion/deletion polymorphism that occurred all of the MA lines. We did find synonymous and non-synonymous mutations unique to individual strains among virus(es)-infected PH-1 strains and several shared polymorphisms consistent with the provenance of each strain. In the overall distribution of synonymous and non-synonymous SNPs, FgV1 genomes with non-synonymous substitutions were more abundant than FgV1 genomes with synonymous substitutions in most generations of MA lines 1, 2, and 4 (Figure 2). MA line 3 had nearly equal non-synonymous and synonymous substitution rates along the entire genome of FgV1 except in the 5^th^ generation. Proportions of non-synonymous vs. synonymous nucleotide substitutions were more variable in MA line 5 than in the other lines. We observed only one polymorphism located in the 5’ UTR region (this was detected in the 1^st^ generation of MA line 4), while the 3’ UTR region seemed highly conserved in all of the MA lines. There were several mutations in MA line 1 (DK21/FgV1) that were not shared with any of the other four MA lines (PH-1/FgV1, PH-1/FgV1 + 2, PH-1/FgV1 + 3, and PH-1/FgV1 + 4), suggesting that evolutionary pathways of the virus could differ depending on the host. For the functional domains in each protein, there were a few mutations in RNA-dependent RNA polymerase (RdRp) and helicase domains, which are generally considered to be important for the survival of RNA viruses (Ranji and Boris-Lawrie, 2010; Choi, 2012).

### Inference of Positive Selection Based on d_N_/d_S_ and LRTs

Because the function of each domain and each ORF in FgV1 has not been clearly identified, we attempted to understand the patterns of spontaneous mutations based on statistical methods including *d_*N*_/d_*S*_* ratios, *pNR/pNC* ratios, and a series of likelihood-ratio tests (LRTs) (Yang, 1998). The models that assume that *d_*N*_/d_*S*_* ratios vary among sites were used to estimate *d_*N*_/d_*S*_* ratios and to detect evidence of positive selection on each codon (Nielsen and Krogh, 1998).

To predict genomic regions under positive selection, we calculated *d_*N*_/d_*S*_* and *pNR/pNC* ratios based on SNPs in each MA line. The altered sequences of each generation of each MA line used for the calculation of *d_*N*_/d_*S*_* and *pNR/pNC* ratios incorporated all of the substitutions from each of three replicates. ORF3 was excluded in this analysis because its low frequency of substitutions is insufficient for analysis by the PAML program. First, the *d_*N*_/d_*S*_* ratios for each MA line and each ORF were calculated using CodeML (Yang, 1997) and were used to infer overall selective pressures on each protein. Based on the assumption that *d_*N*_/d_*S*_* ratios < 1, = 1, and > 1 indicate negative selection, neutral evolution, and positive selection, respectively, there was no clear sign of positive selection in any ORF of any line. Next, positive selection on individual codons within each ORF was inferred by comparing two pairs of models based on LRTs using log-likelihood values for each model. M1 (nearly neutral model) was compared with M2 (positive selection model) and M7 (beta) was compared with M8 (beta & omega > 1) (Table 3). We performed a LRT at significance level α = 0.05 to test the null hypothesis that a simpler model with no consideration of positive selection, i.e., M1 or M7, best fits the observed data. If the LRT statistic is greater than the critical value (5.991 for *P* > 0.05, df = 2), we rejected the null hypothesis and concluded that M2 or M8 fit the observed data significantly better than M1 or M7. As the null hypothesis was rejected only in case of ORF4 of MA line 1 (DK21/FgV1) with the LRT statistics of 6.00 (M1 vs. M2) and 6.01 (M7 vs. M8), respectively, we concluded that the signature of positive selection was found only in the ORF4 of MA line 1. Therefore, determination of the best-fitting models in each ORF and each MA line indicated the signature of positive selection only in the ORF4 of DK21/FgV1.

**TABLE 3:** *d_*N*_/d_*S*_* and determination of best-fitting models based on LRT.

Region^a^	Number of amino acids	MA line	LRT statistic (−2ΔlnL)		Best-fitting model based on LRT		*d_*N*_/d_*S*_* under best-fitting model	
			M1 vs. M2	M7 vs. M8				
ORF1	1550	1	6E-06	–0.05	M1	M7	0.23	0.23
		2	4.38	4.47	M1	M7	0.52	0.60
		3	-6E-06	0.00	M1	M7	0.39	0.39
		4	3.46	3.47	M1	M7	0.57	0.60
		5	0.00	8E-06	M1	M7	0.52	0.52
ORF2	154	1-5	3.11	3.13	M1	M7	0.27	0.30
ORF4	429	1	2.11	2.11	M1	M7	1.00	1.00
		2	6.00	6.01	M2	M8	0.68	0.68
		3	1.39	–1.39	M1	M7	0.71	0.70
		4	0.00	0.00	M1	M7	0.96	0.96
		5	0.01	–0.01	M1	M7	0.59	0.58

*^*a*^−2ΔlnL = the negative of twice the log-likelihood difference between the two models; M1 = model 1, etc.*

### Identification of Positively Selected Sites

To localize specific amino acid sites in ORF4 involved in positive selection during serial passage of DK21/FgV1, we applied the Bayes empirical Bayes (BEB) method in CodeML^6^ (Yang et al., 2005). In the ORF4 of MA line 1 (DK21/FgV1) where positive selection was inferred based on LRTs, the sites predicted to be selected for under M2 and M8 and estimates of parameters for each model are shown in Table 4. In addition, we calculated the allele frequency at each SNP site by calculating the mapped reads with substitution out of total mapped reads at a given site in each sample (Supplementary Table S4). The sites at ORF4 of MA line 1 that were predicted to be under positive selection with the highest posterior probabilities were those with amino acid replacements at 163A (*P* = 0.808 and 0.899 under M2 and M8, respectively) and 289H (*P* = 0.806 and 0.897, respectively), which were not shared in any of the other four MA lines. The replacements at 163A and 289H (nucleotide positions 5772 and 6150, respectively) in the ORF4 sequence brought critical changes in the entire protein structure in the 15^th^ generation of MA line 1, while the structure predicted from the ORF4 sequence of the 10^th^ generation of MA line 1 which only included the replacement at 163A showed no difference in the predicted structure compared that from the wild type. In line with this, the frequency (%) of a mutation at nucleotide position 5772 increased from 0.03449 in the 10^th^ generation to 0.05133 in the 15^th^ generation, along with the emergence of a mutation at nucleotide position 6150 in the 15^th^ generation. The changes in the protein structure caused by replacements at 163A and 289H will further be investigated in the next section to build evidence of positive selection.

**TABLE 4:** Positively selected sites in ORF4 inferred based on CodeML.

Region	Best-fitting model	Parameter estimates under each model	Positively selected sites^a^
ORF4	M2	p0 = 0.94507, p1 = 0.00000, p2 = 0.05493 ω0 = 0.00000, (ω1 = 1.00000), ω2 = 12.3767	**163A (0.808), 289H (0.806)**
	M8	p0 = 0.94506, p = 0.00500, q = 2.86802 (p1 = 0.05494) ω = 12.36550	59V (0.570), 104K (0.554), 152A (0.564), **163A (0.899)**, 234E (0.554), **289H (0.897)**, 356E (0.561), 362A (0.558), 390N (0.549)

*^*a*^Numbers in parenthesis indicate probabilities of predicting each site to be under positive selection. The sites with a probability > 0.8 are in boldface.*

### Inference of Natural Selection Based on the Properties of Amino Acid Replacements

We selected the two most widely used properties to classify amino acids, charge and polarity. After determining the criteria for classification, we calculated *p_*NR*_/p_*NC*_* for ORF1 and ORF4 of each MA line using the methods of Hughes et al. (1990) with the complete genome sequence of Fusarium graminearum virus 1, according to the NCBI database (Accession: NC_006937.2) which was used as a reference. With respect to charge changes, we found that no ORF in any of the five MA lines had a *p*_*NR*_ greater than *pNC*. With respect to polarity, however, *pNR* came close to or even exceeded *p*_*NC*_ in the ORF4 of some generations in all MA lines except MA line 4 (PH-1/V1 + 3) (Table 5). Therefore, natural selection seems to favor polarity changes while conserving the overall charge of amino acids. This result suggests that there was a signature of positive selection in ORF4 with respect to polarity, which had more frequent mutations than ORF1.

**TABLE 5:** *p_*NR*_/p_*NC*_* ratio with respect to charge and polarity.

Property		Charge						Polarity					
Region		ORF1 (*n* = 1550)			ORF4 (*n* = 429)			ORF1 (*n* = 1550)			ORF4 (*n* = 429)		
Line	Gen	*r*	*c*	*r/c*	*r*	*c*	*r/c*	*r*	*c*	*r/c*	*r*	*c*	*r/c*
1	1	0.001	0.001	0.901	0.002	0.004	0.631	0.000	0.001	0.000	0.000	0.005	0.000
	5	0.001	0.001	0.601	0.002	0.005	0.422	0.000	0.002	0.000	0.003	0.005	0.600
	10	0.001	0.002	0.450	0.007	0.009	0.772	0.000	0.002	0.000	0.011	0.006	1.833
	15	0.001	0.001	0.601	0.002	0.005	0.425	0.000	0.002	0.000	0.003	0.005	0.600
2	1	0.000	0.003	0.000	0.000	0.002	0.000	0.001	0.002	0.500	0.000	0.002	0.000
	5	0.000	0.003	0.000	0.002	0.007	0.317	0.001	0.002	0.500	0.003	0.006	0.500
	10	0.001	0.003	0.257	0.002	0.009	0.254	0.001	0.003	0.333	0.006	0.006	1.000
	15	0.001	0.002	0.360	0.002	0.004	0.636	0.001	0.002	0.500	0.003	0.003	1.000
3	2	0.000	0.002	0.000	0.000	0.007	0.000	0.000	0.002	0.000	0.006	0.003	2.000
	6	0.001	0.003	0.258	0.000	0.005	0.000	0.001	0.003	0.333	0.003	0.003	1.000
	11	0.000	0.003	0.000	0.000	0.005	0.000	0.000	0.003	0.000	0.003	0.003	1.000
	12	0.000	0.003	0.000	0.000	0.005	0.000	0.000	0.003	0.000	0.003	0.003	1.000
4	2	0.001	0.002	0.328	0.002	0.004	0.631	0.000	0.003	0.000	0.000	0.005	0.000
	6	0.001	0.002	0.328	0.000	0.004	0.000	0.000	0.003	0.000	0.000	0.005	0.000
	11	0.001	0.003	0.241	0.000	0.004	0.000	0.001	0.003	0.333	0.000	0.005	0.000
	16	0.001	0.003	0.240	0.000	0.004	0.000	0.001	0.003	0.333	0.000	0.005	0.000
5	2	0.000	0.003	0.000	0.000	0.004	0.000	0.000	0.003	0.000	0.000	0.003	0.000
	6	0.000	0.003	0.000	0.000	0.004	0.000	0.000	0.003	0.000	0.000	0.003	0.000
	11	0.000	0.003	0.000	0.002	0.004	0.628	0.000	0.003	0.000	0.003	0.003	1.000
	12	0.000	0.004	0.000	0.002	0.004	0.628	0.000	0.004	0.000	0.003	0.003	1.000

*Line: MA line; Gen: generation; *n*: number of amino acids; *r*: *p*_*NR*_; *c*: *p*_*NC*_.*

### Comparison of Predicted Protein Structures

**FIGURE 3:**
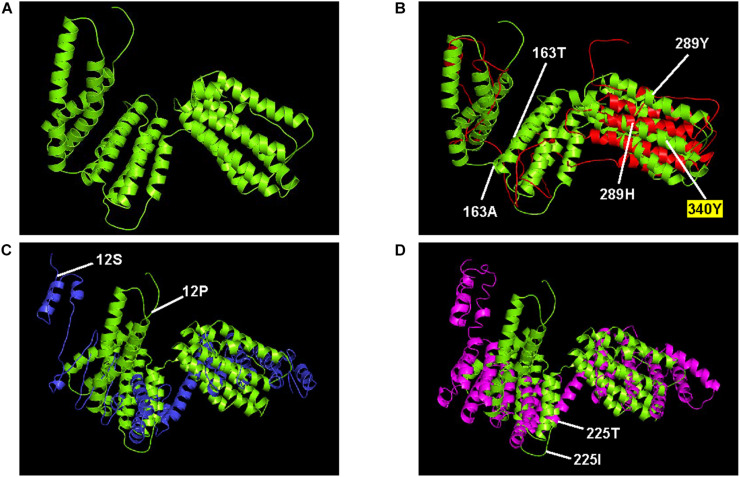
ORF4 protein structures that were substantially changed during serial passage. The protein structure predicted from **(A)** the wild type (lime) are aligned with the structures predicted from **(B)** 15^th^ generation of MA line 1 (red), **(C)** the 10th generation of MA line 2 (blue), and **(D)** the 2^nd^ generation of MA line 3 (magenta). Also indicated are amino acid replacements predicted to change the entire structure of the protein (163, 289, 12, and 225), and the new active site for enzyme binding (340).

Protein structures for ORFs 1, 2, 3, and 4 with 18, 4, 3, and 17 amino acid substitution mutations, respectively, were compared. Each amino acid replacement was assigned to one of three groups based on the degree of change in protein structure (Table 6). For ORF1, all of the replacements caused minor changes in the protein structure and caused no changes in the ligand or enzyme-binding sites. For ORF2, although some replacements (41G→S, 41G→V) resulted in changes to the entire structure of the protein, the replacements were found in only one of three replicates in a generation, which may represent a low possibility of fixation in the population. For ORF3, only three replacements were found, and these did not result in any change in the secondary structure of the protein. For ORF4, replacements in four amino acid sites including 163A and 289H, which were predicted to be under positive selection, seemed to cause changes in the entire protein structure. When there were replacements at 163A and 289H in the ORF4 of MA line 1, the alpha helix structures covering about half of the protein at the N-terminal part were modified into coil structures (Figure 3, panel A for the wild type and panel B for imposed). This modification generated a new active site for enzyme binding (340Y) and slight alterations in ligand binding sites, which seems to indicate the addition of a function to the protein with the original functions maintained. Also for ORF4, there were replacements at 12P in MA line 2 and at 225I in MA line 3 that resulted in changes to the entire structure of the protein (Figure 3, panels C and D). Unlike the replacements found in MA line 1, however, these replacements caused critical omissions in ligand binding sites and did not add new active sites. In addition, these replacements were not found in subsequent generations in each line, which might indicate that they had negative effects on the fitness of the virus and were therefore purged by purifying selection.

**TABLE 6:** Properties of amino-acid replacements based on changes in protein structures.

	Region and degree of change^a^								
Region	ORF1			ORF2			ORF4		
Degree^a^	1	2	3	1	2	3	1	2	3
Sites	251E→G 266V→A 314G→A 382T→S 392H→N 731P→S 782N→S 883M→L 948A→V 978S→P 1020L→F 1246A→D 1316V→I	616D→A 865A→V 975A→T 1005I→V 1490I→M	None	None	31L→S 80E→G	41G→S 41G→V	24F→L 57A→D 59V→L 101D→V 104K→R 152A→S 234E→K 362A→T 390N→D	108T→A 227D→G 323E→D 356E→V	12P→S 163A→T 225I→T 289H→Y

*^*a*^ Degree of change: Amino acid replacements cause (1) no change in the structure of the protein; (2) a transition between the alpha helix and coil, which does not change the entire structure of a protein; or (3) changes in the entire structure of a protein.*

According to the NCBI database, ORF4 protein of FgV1 had the highest sequence similarity (27.2%) with a bacterial SMC-like (structural maintenance of chromosomes) domain (GenBank conserved domain TIGR02168) and with homologous proteins of other putative fusariviruses (Hrabáková et al., 2017), including ORF2 of Penicillium roqueforti ssRNA mycovirus 1 which showed the highest amino acid sequence identity (27.15%). As the SMC domain has been detected in similar positions on ORF2 (ORF4 in the case of FgV1) protein in most fusariviruses, the significance and function of this domain can be inferred from the function of the bacterial SMC domain, which, in prokaryotes, is proposed to be involved in the repair of DNA double-strand breaks (DSBs) and the maintenance of genome integrity (Pellegrino et al., 2012).

### Relationship Between Viral Fitness and the Number of Mutations

Taking into account the selective pressures predicted based on *d_*N*_/d_*S*_* ratios and also the inferred presence of positively selected sites, we assumed that most of the mutations were neutral or deleterious. Based on this assumption, we expected that the fitness of FgV1 would gradually decline during passages. The fitness of the virus was estimated using two methods, i.e., real-time qRT-PCR, and the enumeration of viral RNA reads.

**FIGURE 4:**
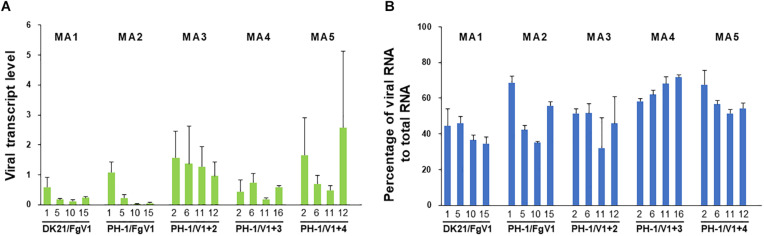
Fitness of FgV1 estimated by **(A)** real-time PCR and **(B)** numbers of mapped viral reads. Numbers below each lane indicate the generations of each MA line.

**FIGURE 5:**
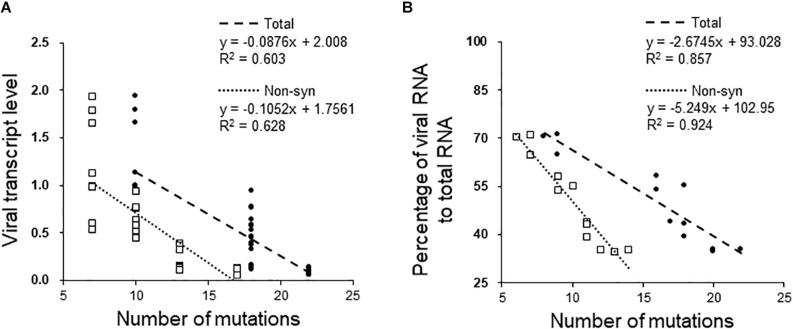
Effects of mutations acquired by passaging on viral fitness in the MA2 line. Relationship between the fitness of FgV1 estimated by **(A)** real-time qRT-PCR and by **(B)** counting the number of mapped reads, and the number of total and non-synonymous mutations. Dotted and dashed lines indicate the linear regression between the fitness and the number of total (•) and non-synonymous (□) mutations, respectively.

Figure 4 shows the fitness of FgV1 in each of four generations of the five MA lines. The results obtained by two methods did not exactly correspond to one another, but a similar pattern was found among some MA lines. In MA lines 1, 2, and 5, the fitness declined at least up to the 10th generation despite a sudden increase in the 15th generation of MA line 5 based on real-time qRT-PCR (Figure 4A) and in the MA line 2 based on the number of viral reads (Figure 4B). To determine whether the decline was related to the accumulation of deleterious mutations, the relationship between the fitness and the number of total or non-synonymous mutations was analyzed. First, we determined whether there was a linear relationship between the fitness and the number of total mutations; a negative linear relationship between the two fitness variables and the number of mutations was found only in MA line 2 (PH-1/FgV1) (Figure 5). When the number of non-synonymous mutations were used instead of total mutations, we obtained the same result but with slightly higher R^2^ values (0.628 and 0.924). Next, we conducted correlation analyses to quantify the strength of the negative linear relationship, which confirmed the negative correlation between the fitness and the number of total mutations (correlation coefficient, *r* = −0.78 and −0.93, respectively, for the above two methods) and non-synonymous mutations (*r* = −0.76 and −0.96, respectively). Overall, the results suggest that although the total number of mutations may help explain the decline in viral fitness in MA line 2 (PH-1/FgV1), factors other than the total number of mutations appear to explain the decline in FgV1 fitness for MA lines 1 and 5.

## Discussion

This work represents a first study of fitness changes of a mycovirus caused by the accumulation of spontaneous mutations. When FgV1 was unaffected by other co-infecting viruses, we found that changes in FgV1 fitness during serial passage were dominated by neutral or deleterious mutations. This result is in agreement with previous findings from many other RNA viruses including bacteriophage φ6, VSV, FMDV, and HIV-1 (Chao, 1990; Duarte et al., 1992; Escarmís et al., 1996; Yuste et al., 1999). Despite biological differences among the virus-host systems, observed decreases in viral fitness seem to support the operation of Muller’s ratchet under repeated bottleneck passages. We did also find evidence of positive selection on a few sites within the ORF4 of FgV1 as indicated by maximum-likelihood methods and predicted protein structures. Interestingly, several mutations in MA line 1 (DK21/FgV1) were not shared in any of the other four MA lines, including mutations at nucleotide positions 5772 and 6150 (with average mutation frequency percentage of 0.05133 and 0.04979, respectively, in three biological replicates of the 15^th^ generation) which bring amino acid replacements at 163A→T and 289H→Y of ORF4 which were predicted to be under positive selection. This implies the possibility of different cellular mechanisms or immune responses of DK21 (*F. boothii*; lineage 3) and PH-1 (*F. graminearum*, lineage 7) against pathogens, which can be inferred, for instance, from the significantly high self-fertility of the strains of lineage 7 (Lee et al., 2012). Similarly, in the previous study on VSV under Muller’s ratchet, bottleneck passages on a new host cells led to more regular and severe fitness losses than passages on the cells to which the virus strain used in the study had been well adapted for years (Duarte et al., 1992). In the present study, we found that a negative linear relationship between the fitness of FgV1 and the number of mutations was found only in PH-1, between two strains (DK21 and PH-1) infected singly with FgV1 and free from other co-infecting viruses. Assuming that FgV1 had been well adapted to its natural host, DK21, we can expect that negative effects of deleterious mutations which cause fitness losses will stand out in the natural host rather than in the laboratory host. Despite the high mutation rates of RNA viruses which contribute to their remarkable ability to adapt to fluctuating environments, this study and the previous studies found the great possibility of fitness decrease during repeated bottleneck events as a consequence of Muller’s ratchet.

### Factors Affecting Evolution—Mutational Robustness

One factor that might affect the evolution of a virus is its ability to deal with accumulated deleterious mutations, which account for a large proportion of mutations in a small population with severe bottlenecks. Such an ability, which is termed mutational robustness, is defined as constancy of phenotype in the face of mutational perturbation and is critical for the adaptation of viruses to changing environments. In an MA experiment using the RNA virus phage φ6, researchers determined that variance in fitness was significantly lower for lines that evolved under lower levels of co-infection only in the short term (Montville et al., 2005). In the long term, however, a defective virus can buffer the negative effects of mutations through frequent genetic complementation at a high level of co-infection, which eventually enables defective viruses to propagate and thus decreases mutational robustness (Lauring et al., 2013). In this regard, the decreased robustness of viruses which obtained high levels of accumulated mutations under high levels of co-infection might explain the large variation in viral loads and host phenotypes among replicates in the 11th and 12th generations of PH-1/FgV1 + 2 (Figures 2, 5 and Supplementary Figure S3). This seems more convincing if one considers that the levels of viral RNA accumulation in the host fungi were higher for FgV1 and 2 than for FgV3 and FgV4.

### Factors Affecting Evolution—Interaction Between Viruses

Within-host interactions between plant viruses have been widely studied. When two or more viruses infect the same host at the same time or within short intervals, the fitness of each virus depends not only on its own ability to adapt to the environment, but also on the activity of the other co-infecting virus(es). Unrelated viruses generally interact with each other in a synergistic manner, resulting in higher accumulations of the viruses and more severe symptoms in their hosts (Syller and Grupa, 2016). A synergistic interaction between mycoviruses has also been observed in a pathosystem involving *Sclerotinia sclerotiorum* and a hypovirulence-associated mycoreovirus, Sclerotinia sclerotiorum mycoreovirus 4 (SsMYRV4). In the latter case, SsMYRV4 suppressed non-self-recognition of its host by suppressing G-protein signaling pathways and thereby facilitated horizontal transmission of heterologous viruses between two fungal host strains that were otherwise vegetatively incompatible (Wu et al., 2017). Antagonistic interactions between co-infecting viruses, however, also occur and involve cross-protection and mutual exclusion (Syller and Grupa, 2016). Mutual exclusion has been frequently found in human parasites. With dengue virus, for example, virus titers and virus transmission were lower with infections by multiple virus strains than with infections by single virus strains (Pepin et al., 2008). The effects of interactions between FgV1 and the other co-infecting virus were not rigorously determined in the current study. Isolates of MA line 3 (PH-1/FgV1 + 2) showed unstable phenotypes and decreased vertical transmission of FgV1. In case of MA line 4 and 5 (PH-1/FgV1 + 3 and 1 + 4), however, the transmission of FgV1 was maintained, while the transmission of FgV3 and FgV4 decreased with fluctuations between generations. As demonstrated in the previous study on HIV-1, one of several mechanisms which may accelerate fitness losses induced by bottleneck passages is the level of virus titers. Titers from HIV-1 plaques, which were relatively lower than those from φ6, VSV and FMDV, seems to have further reduced the possibility of competition and compensatory mutations among quasispecies and resulted in relatively large fitness losses (Yuste et al., 1999; De la Iglesia and Elena, 2007). Besides, repeated transfers of large virus populations, represented by high level of virus titers, increased the fitness of the populations (Clarke et al., 1993). Since the numbers of viral genomes of FgV1 do not distinctly vary among MA lines 3, 4 and 5 in our pathosystem, we can expect that the ability of FgV1 to adapt to the environment would be influenced by other factors related to interactions between viruses, rather than the aforementioned possibility of competition, etc. When it comes to interactions between viruses, our previous study revealed that the dsRNA accumulation of FgV2 or FgV3 was higher in fungal transformed mutants expressing FgV1−encoded ORF2 than wild-type strains, because FgV1-encoded pORF2 was able to inhibit the host’s antiviral RNA silencing responses by suppressing the transcription of FgDICER2 and FgAGO1 (Yu et al., 2020). Therefore, we expected that FgV1 might help accumulation or maintenance of co-infecting viruses, as shown by the synergistic effect of Cryphonectria hypovirus 1 (CHV1) on a co-infecting dsRNA virus, mycoreovirus 1 (MyRV1), represented by increased accumulation and vertical transmission of MyRV1 (Sun et al., 2006). The effect of interactions between FgV1 and the other co-infecting virus or the reason for aforementioned changes in the phenotype of MA line 4 has not been rigorously determined in the current study. However, tendency in dsRNA accumulation among biological replicates in the same generation of MA line 3 may support our assumption that there is an antagonistic, rather than synergistic, interaction between FgV1 and FgV2 (Supplementary Figure S1). In case of MA line 4 and 5, there was no evidence of beneficial effects of FgV1 on the vertical transmission or accumulation of its counterpart. On the contrary, especially in case of FgV4, its accumulation was often below the significant detection level in RT-PCR, which was not the case of isolates infected singly with FgV4 (Supplementary Figure S2), despite average vertical transmission ratio of around 60% during passaging of MA line 5 (data not shown). However, there is a possibility that FgV1-infected fungal host does not provide a favorable condition for FgV3 and FgV4 despite the host’s antiviral activities inhibited by FgV1. Because we focused on the adaption and fitness of FgV1 in this study, further investigations are needed to clarify the effects of interactions between FgV1–4.

### Effects of Host Species on the Evolution of Viruses

One concept widely used to evaluate evolutionary processes in a population is the distribution of fitness effects (DFE), which explains the proportion of new mutations that are advantageous, neutral, and deleterious and whether and how those effects alter the total fitness of the population. The shape of the DFE curve varies depending on species, population size, and other factors (Eyre-Walker et al., 2006). In a study of tobacco etch virus infecting eight susceptible host species, the characteristics of the fitness effects of mutations differed between the natural host (along with its close relative) and the alternative hosts (Lalić et al., 2011). DFE therefore indicates the likelihood that a pathogen can cross the species barrier and successfully infect a new host (Lalić et al., 2011). The results of the current study suggest that the evolutionary trajectories of FgV?/FgV1–4? may differ among the lineages of FGSG. Both of the *F. graminearum* strains used in this study, DK21 (*F. boothii*; lineage 3) and PH-1 (*F. graminearum sensu stricto*; lineage 7), not only belong to the three most closely related lineages of FGSG, i.e., *F. graminearum*, *F. boothii*, and *F. asiaticum*, but also to the four lineages found to date in South Korea, i.e., *F. graminearum* (representing 75% of all isolates), *F. boothii* (12%), *F. asiaticum* (12%), and *F. meridionale* (1%) (Lee et al., 2012). In terms of their origin relative to plant hosts, however, *F. graminearum* mostly infects maize and is thought to have been introduced from the United States, while *F. boothii* seems to have originated from local populations infecting rice (Lee et al., 2012). It follows that despite the close phylogenetic relationship between PH-1 and DK21, their difference in host preference may be related to their differences in supporting spontaneous mutations of FgV1 and may also help explain why FgV1 has adapted so well to the laboratory environment.

A new asymptomatic strain FgV1-ch was recently isolated and characterized in China (Zhang et al., 2020). Interestingly, FgV1-ch did not show strong virulence to the host (PH-1), despite slight differences in the colony morphology, mycelial growth rate, and the production of conidia between FgV1-ch-infected and virus-free strains of PH-1 (Zhang et al., 2020). Regarding its sequence, FgV1-ch showed 95.91% nucleotide identities (6350/6621) with the reference sequence of FgV1 (Accession number; NC_006937.2) in the NCBI database, with 26 amino acid replacements (Figure 2). We found significant variation between the numbers of synonymous and non-synonymous substitutions in FgV1-ch as well as differences in the numbers of substitutions between FgV1-ch and the MA lines in the current study. With respect to the number of substitutions, one study with RNA and DNA viruses found a decline in the ratio of transitions to transversions (Ts/Tv) over time (Duchêne et al., 2015); the authors suggested that the decline could be caused by an underestimation of the Ts/Tv ratio due to the nature of RNA viruses, which rapidly attain mutational saturation. Likewise, variable genes might attain saturation more rapidly and thus display a stronger decline in Ts/Tv than conserved genes (Duchêne et al., 2015). In this context, the lower Ts/Tv ratio in ORF1 (6.9) than in ORF4 (11.0) along with the relatively higher concentration of substitutions in ORF1 (212 substitutions of 4653 nt) than in ORF4 (36 of 1290 nt) in FgV1-ch (Zhang et al., 2020) might reflect a rapid mutational saturation in ORF1 in the course of the evolution of the asymptomatic strain FgV1-ch in a different strain of the host fungus.

In a previous study, FgV1 was artificially transmitted via protoplast fusion to filamentous fungi of two different genera, *Cryphonectria parasitica* and *Fusarium oxysporum*, and was found to maintain its ability to induce hypovirulence in those species (Lee et al., 2014). Whether FgV1 can adapt to the more stringent environments in non-host species is an important question generated by the current study. Further comparisons of the genetic variation of FgV1 in non-hosts and natural hosts and of the modifications in the gene expression of those FgV1-infected hosts are needed to increase our understanding of factors affecting viral pathogenicity and evolution.

## Data Availability Statement

The datasets generated for this study can be found in the online repositories. The virus and virus-like sequences derived from this study can be found in GenBank under the accession number PRJNA656941.

## Author Contributions

J-IH and K-HK designed the experiments, analyzed the data, and wrote the manuscript. J-IH, JY, and HC performed the experimental work. All authors contributed to the article and approved the submitted version.

## Conflict of Interest

The authors declare that the research was conducted in the absence of any commercial or financial relationships that could be construed as a potential conflict of interest.

## Footnotes

1 http://ugene.net/

2 http://ugene.net/

3 http://igem.temple.edu/labs/nei/

4 http://zhanglab.ccmb.med.umich.edu/I-TASSER/

5 https://pymol.org/2/

6 http://abacus.gene.ucl.ac.uk/software/paml.html
