# Supplementary material for: The Signatures of Natural Selection and Molecular Evolution in Fusarium graminearum Virus 1
Source: Front Microbiol. 2020 Nov 12;11:600775. doi: 10.3389/fmicb.2020.600775 (PMC7688778; doi:10.3389/fmicb.2020.600775)
Supplement: Supplementary file 1 [file Table_1.DOCX]

**Supplementary Table S1.** List of primers used in real-time PCR and semi-quantitative PCR

| **Primer** | **Sequence** | **Target** | **Purpose** |
| --- | --- | --- | --- |
| FgV1_1632F  FgV1_1786R | 5’- ACTATCATTGGTACGCCGCTTGTT-3’  5’- CAGCGCGAATGCCTAAAATACAG-3’ | Fusarium graminearum virus 1 | To estimate the accumulation of FgV1 via real-time PCR |
| FgV3_7422F  FgV3_7624R | 5’-CTTAGTTCACGGCGAGTATTTCAA-3’  5’-CTAATTCCGGTGGTACAGTGGTC-3’ | Fusarium graminearum virus 3 | To confirm the infection of FgV3 in MA line 4 via semi-quantitative PCR |
| FgV3_RT_RV | 5’-TTCTTCTTGGAGAATCTTAATCC-3’ | Fusarium graminearum virus 3 | To reverse-transcribe mRNA of FgV3 for semi-quantitative PCR |
| FgV4_R1_1311F  FgV4_R1_1761R | 5’-ATGGGGTGGTTCAAAGGCAATGT-3’  5’-CCTGCTCCACGGAAATACCCTCTA-3’ | RNA1 of Fusarium graminearum virus 4 | To confirm the infection of FgV4 in MA line 5 via semi-quantitative PCR |
| EF1_RT_F  EF1_RT_R | 5’-ATGGGGTGGTTCAAAGGCAATGT-3’  5’-CCTGCTCCACGGAAATACCCTCTA-3’ | Translation elongation factor 1-alpha gene (EF1-α) of *Fusarium graminearum* | To estimate the expression of a reference gene as an internal control in real-time PCR |
| UBH_RT_F  UBH_RT_R | 5’-ATGGGGTGGTTCAAAGGCAATGT-3’  5’-CCTGCTCCACGGAAATACCCTCTA-3’ | Ubiquitin C-terminal hydrolase gene (UBH) of *Fusarium graminearum* | To estimate the expression of a reference gene as an internal control in real-time PCR |
