# Supplementary material for: The Signatures of Natural Selection and Molecular Evolution in Fusarium graminearum Virus 1
Source: Front Microbiol. 2020 Nov 12;11:600775. doi: 10.3389/fmicb.2020.600775 (PMC7688778; doi:10.3389/fmicb.2020.600775)
Supplement: Supplementary file 2 [file Table_2.DOCX]

**Supplementary Table S2.** Percentage of the mapped FgV1 viral reads in total RNA samples. This data supports the high level of FgV1 viral loads in the host. HISAT2 (version 2.1.1) and BBMap programs were used in the alignment of RNA sequencing reads.

| **Program** | | **HISAT2** | | | **BBMap** | | |
| --- | --- | --- | --- | --- | --- | --- | --- |
| **MA Line** | **Gen** | **Rep 1** | **Rep 2** | **Rep 3** | **Rep 1** | **Rep 2** | **Rep 3** |
| **1** | **1** | 54.46 | 43.08 | 35.55 | 54.75 | 43.33 | 35.74 |
|  | **5** | 42.33 | 46.19 | 49.24 | 42.49 | 46.34 | 49.42 |
|  | **10** | 35.36 | 39.52 | 34.39 | 35.49 | 39.68 | 34.50 |
|  | **15** | 30.12 | 35.96 | 36.54 | 30.35 | 36.21 | 36.78 |
| **2** | **1** | 70.76 | 64.45 | 69.96 | 71.04 | 64.65 | 70.26 |
|  | **5** | 43.79 | 39.17 | 42.94 | 43.99 | 39.34 | 43.10 |
|  | **10** | 35.07 | 34.45 | 35.21 | 35.21 | 34.61 | 35.38 |
|  | **15** | 57.69 | 53.43 | 54.72 | 58.08 | 53.76 | 55.04 |
| **3** | **2** | 52.78 | 52.29 | 48.09 | 53.08 | 52.62 | 48.32 |
|  | **6** | 45.22 | 54.80 | 53.83 | 45.51 | 55.20 | 54.13 |
|  | **11** | 15.78 | 30.72 | 49.20 | 15.88 | 30.90 | 49.51 |
|  | **12** | 51.25 | 56.78 | 28.77 | 51.51 | 57.17 | 28.94 |
| **4** | **2** | 57.08 | 47.74 | 59.50 | 57.47 | 48.04 | 59.87 |
|  | **6** | 60.20 | 57.65 | 64.33 | 60.54 | 57.96 | 64.74 |
|  | **11** | 69.73 | 68.74 | 63.03 | 70.19 | 69.21 | 63.55 |
|  | **16** | 70.06 | 66.08 | 72.73 | 70.64 | 66.59 | 73.33 |
| **5** | **2** | 74.98 | 68.09 | 58.97 | 75.30 | 68.34 | 59.21 |
|  | **6** | 57.29 | 57.67 | 54.14 | 57.51 | 57.93 | 54.30 |
|  | **11** | 49.40 | 53.71 | 50.49 | 49.57 | 53.98 | 50.69 |
|  | **12** | 56.14 | 55.16 | 50.93 | 56.37 | 55.40 | 51.09 |
