# Supplementary material for: The Signatures of Natural Selection and Molecular Evolution in Fusarium graminearum Virus 1
Source: Front Microbiol. 2020 Nov 12;11:600775. doi: 10.3389/fmicb.2020.600775 (PMC7688778; doi:10.3389/fmicb.2020.600775)
Supplement: Supplementary file 5 [file Data_Sheet_1.DOCX]

# Supplementary Figure


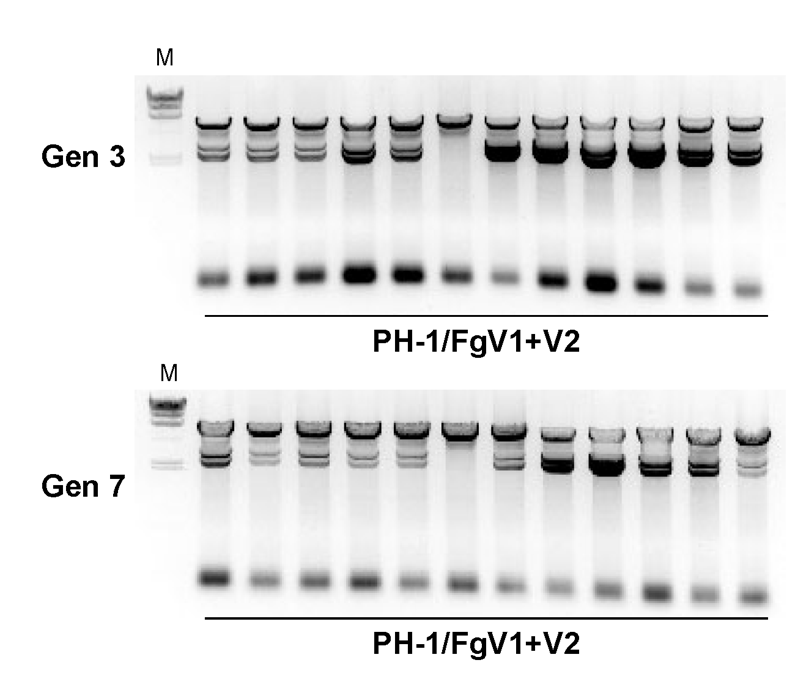


**Supplementary Figure S1.** Agarose gel (0.8%) electrophoresis of viral dsRNA. 30µg of total RNA from FgV1 and 2-infected *Fusarium graminearum* PH-1 strain were used following DNaseI and S1 nuclease treatment. Different accumulation level of FgV1 and FgV2 dsRNA among biological replicates in the 3^rd^ (upper panel) and 7^th^ generation (lower panel). The largest band in whole lanes represents dsRNA of FgV1 (6.6 kb), while smaller segmented bands represent dsRNAs from FgV2 (2.4 - 3.6 kb). Lane M, lambda DNA digested with *Hin*dⅢ.

# Supplementary Figure


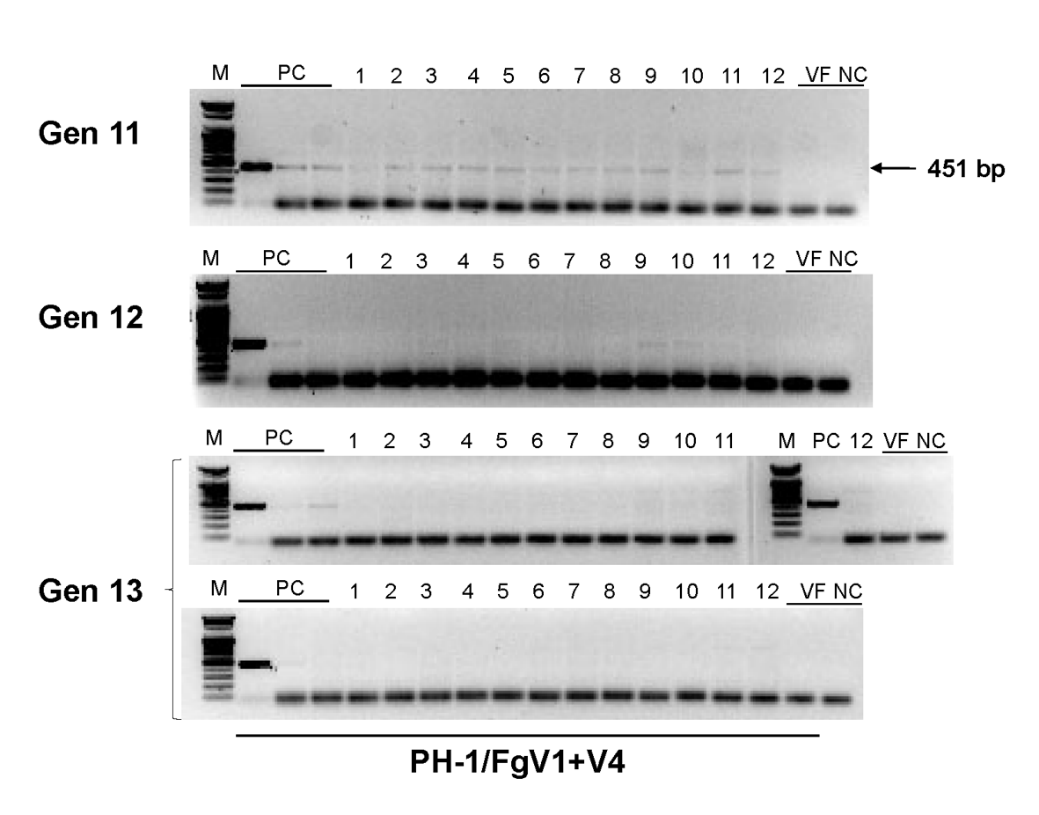


**Supplementary Figure S2.** Detection of FgV4 in FgV1 and 4-infected *Fusarium graminearum* PH-1 strain by RT-PCR. The target PCR products of FgV4 (after 35 amplification cycles) and visualized on a 1.2% agarose gel under UV lights. Amplified fragment sizes are indicated to the right of the image. Lane 1-12 represent single conidial isolates of each generation (indicated number left of the image). Lane M, 100bp DNA ladder (Bioneer).

# Supplementary Figure

**
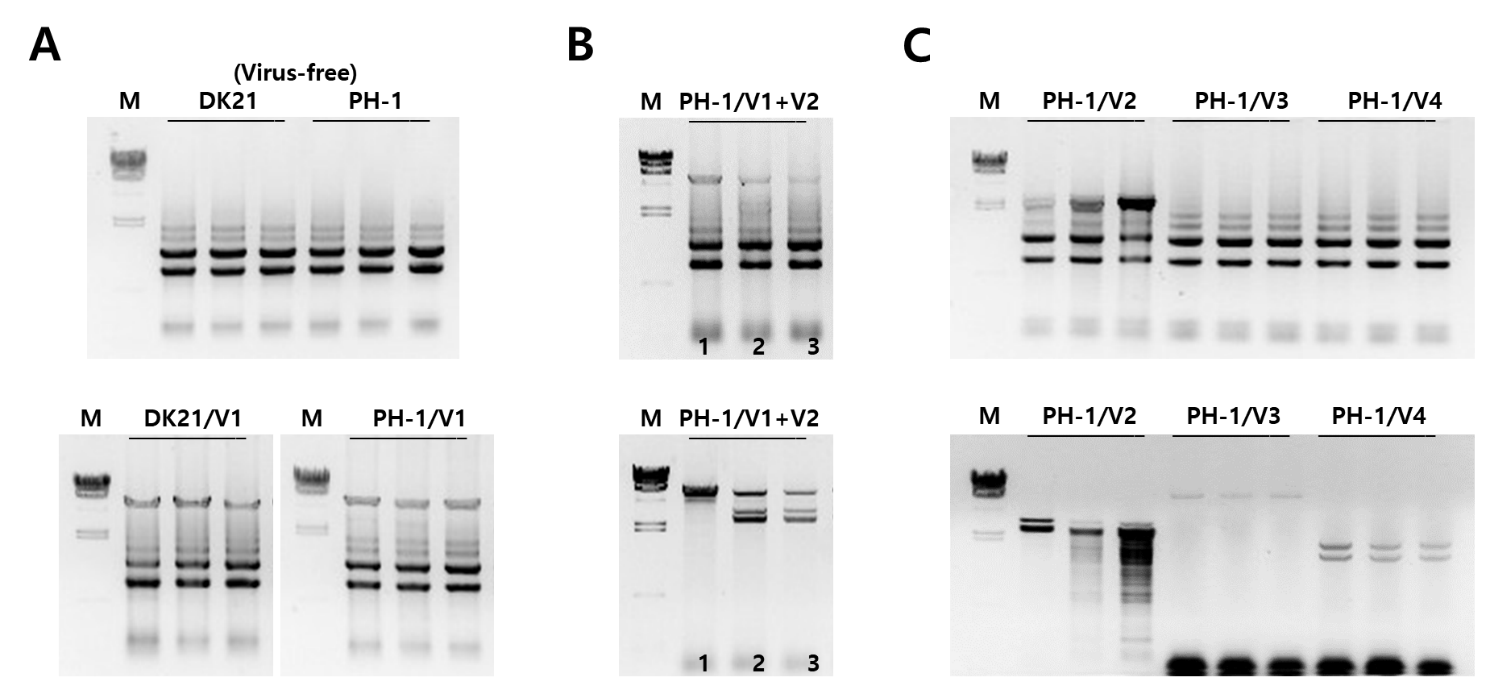
**

**Supplementary Figure S3.** Agarose gel (0.8%) electrophoresis of total RNA of fungi and viral dsRNA. (A) Total RNA (3µg) of virus-free or FgV1-infected *Fusarium graminearum* DK21 and PH-1. The dominant band in the FgV1-infected samples represents the full-length viral dsRNA (6.6 kb), while smaller bands represent fungal rRNA molecules. (B) Total RNA (3µg) of FgV1-infected (lane 1) and FgV1 and 2-infected (lane 2 and 3) PH-1 (upper panel), and dsRNA of FgV1 (lane 1) and FgV1 and 2 (lane 2 and 3) isolated from 10µg of total RNA through DNaseI and S1 nuclease treatment (lower panel). In the lower panel, the largest band in lane 1-3 represents dsRNA of FgV1 (6.6 kb), while smaller bands in lane 2 and 3 represent dsRNA segments of FgV2 (2.4 - 3.6 kb). (C) Total RNA (3µg) of FgV2, 3 or 4-infected PH-1 (upper panel), and dsRNA of FgV2, 3 and 4 (lower panel). 10µg of total RNA of PH-1/FgV2 and 30µg of total RNA of PH-1/FgV3 and 4 were treated with DNaseI and S1 nuclease treatment. Relative viral loads in the host can be inferred from the dsRNA band intensity and the amount of total RNA used for dsRNA isolation. Lane M, lambda DNA digested with *Hin*dⅢ.

# 
